# Supplementary material for: Surface color and predictability determine contextual modulation of V1 firing and gamma oscillations
Source: eLife. 2019 Feb 4;8:e42101. doi: 10.7554/eLife.42101 (PMC6391066; doi:10.7554/eLife.42101)
Supplement: Transparent reporting form [file elife-42101-transrepform.docx]

***eLife’s* transparent reporting form**

We encourage authors to provide detailed information *within their submission* to facilitate the interpretation and replication of experiments. Authors can upload supporting documentation to indicate the use of appropriate reporting guidelines for health-related research (see [EQUATOR Network](http://www.equator-network.org/%20)), life science research (see the [BioSharing Information Resource](https://biosharing.org/)), or the [ARRIVE guidelines](http://www.plosbiology.org/article/info:doi/10.1371/journal.pbio.1000412) for reporting work involving animal research. Where applicable, authors should refer to any relevant reporting standards documents in this form.

If you have any questions, please consult our Journal Policies and/or contact us: [editorial@elifesciences.org](mailto:editorial@elifesciences.org).

**Sample-size estimation**

- You should state whether an appropriate sample size was computed when the study was being designed
- You should state the statistical method of sample size computation and any required assumptions
- If no explicit power analysis was used, you should describe how you decided what sample (replicate) size (number) to use

Please outline where this information can be found within the submission (e.g., sections or figure legends), or explain why this information doesn’t apply to your submission:

We did not use an explicit power analysis when determining the number of animals or sessions.

Two rhesus macaques were used. This number reflects the standards of the field, which attempts to minimize the number of animals used while keeping a second animal for replication, to ensure that the findings do not reflect particular idiosyncrasies of one animal.

Statistical analyses are all performed either across trials within a session in the case of single-session examples (in some Supplementary Figures) or across trials and sessions. The number of trials per condition and sessions were based our own pilot data, showing effect sizes large enough to see effects in individual sessions, as well as the typical trial number completed per animal on a day. The number of sessions conducted per animal was determined in part by implant viability.

**Replicates**

- You should report how often each experiment was performed
- You should include a definition of biological versus technical replication
- The data obtained should be provided and sufficient information should be provided to indicate the number of independent biological and/or technical replicates
- If you encountered any outliers, you should describe how these were handled
- Criteria for exclusion/inclusion of data should be clearly stated
- High-throughput sequence data should be uploaded before submission, with a private link for reviewers provided (these are available from both GEO and ArrayExpress)

Please outline where this information can be found within the submission (e.g., sections or figure legends), or explain why this information doesn’t apply to your submission:

The data was collected over the course of several weeks for each animal. One or several sessions were collected each day depending on how many trials the animals completed before stopping.

Information about session and trial numbers are provided in the figure legends. The number of selected sites is mentioned in the text where appropriate. Data (in particular site selection) selection criteria are described in the Methods section.

**Statistical reporting**

- Statistical analysis methods should be described and justified
- Raw data should be presented in figures whenever informative to do so (typically when N per group is less than 10)
- For each experiment, you should identify the statistical tests used, exact values of N, definitions of center, methods of multiple test correction, and dispersion and precision measures (e.g., mean, median, SD, SEM, confidence intervals; and, for the major substantive results, a measure of effect size (e.g., Pearson's r, Cohen's d)
- Report exact p-values wherever possible alongside the summary statistics and 95% confidence intervals. These should be reported for all key questions and not only when the p-value is less than 0.05.

Please outline where this information can be found within the submission (e.g., sections or figure legends), or explain why this information doesn’t apply to your submission:

The detailed description of the statistical analysis can be found in the methods and results sections, we largely performed nonparametric tests based on bootstrapping or permutation. The multiple comparison correction as well as dispersion measures are described in the methods. Effect sizes are reported as fold-changes from baseline or between conditions, or in Hz for frequency measures. The model fits are evaluated using R-squared (explained variance). The p-values are reported in the figure legends. P-values for non-significant results are reported with their precise value.

Note that the bootstrapping procedure employed with N=1000 bootstraps and a two-sided test does not allow for greater precision of p-values than p<0.002 before multiple comparison correction, and that the data allow for a good visualization of effect sizes and dispersion measures. We therefore report p-values as <0.05 two-sided and multiple-comparison corrected, with the exception of parametric tests.

(For large datasets, or papers with a very large number of statistical tests, you may upload a single table file with tests, Ns, etc., with reference to sections in the manuscript.)

**Group allocation**

- Indicate how samples were allocated into experimental groups (in the case of clinical studies, please specify allocation to treatment method); if randomization was used, please also state if restricted randomization was applied
- Indicate if masking was used during group allocation, data collection and/or data analysis

Please outline where this information can be found within the submission (e.g., sections or figure legends), or explain why this information doesn’t apply to your submission:

There was no group allocation, trials within a session were grouped based on their condition.

**Additional data files (“source data”)**

- We encourage you to upload relevant additional data files, such as numerical data that are represented as a graph in a figure, or as a summary table
- Where provided, these should be in the most useful format, and they can be uploaded as “Source data” files linked to a main figure or table
- Include model definition files including the full list of parameters used
- Include code used for data analysis (e.g., R, MatLab)
- Avoid stating that data files are “available upon request”

Please indicate the figures or tables for which source data files have been provided:

The datasets underlying the figures are made available on Dryad upon acceptance of the manuscript.
